# Crystal and mol­ecular structures of di­chlorido­palladium(II) containing 2-methyl- or 2-phenyl-8-(diphenyphosphan­yl)quinoline

**DOI:** 10.1107/S2056989020016096

**Published:** 2021-01-01

**Authors:** Masatoshi Mori, Atsushi Namioka, Takayoshi Suzuki

**Affiliations:** a Graduate School of Natural Science and Technology, Okayama University, Okayama, 700-8530, Japan; b Research Institute for Interdisciplinary Science, Okayama University, Okayama, 700-8530, Japan

**Keywords:** 8-quinolylphosphane, square-planar coordination, tetra­hedral distortion, inter­molecular stacking inter­action., crystal structure

## Abstract

The crystal structures of di­chlorido­palladium(II) complexes bearing 2-methyl- and 2-phenyl-8-(di­phenyl­phosphan­yl)quinoline, namely, di­chlorido­[8-(di­phenyl­phosphan­yl)-2-methyl­quinoline-κ^2^ *N*,*P*]palladium(II), [PdCl_2_(C_22_H_18_NP)] (**1**) and di­chlorido­[8-(di­phenyl­phosphan­yl)-2-phenyl­quinoline-κ^2^ *N*,*P*]palladium(II), [PdCl_2_(C_27_H_20_NP)] (**2**), were analyzed and compared to that of the 8-(di­phenyl­phosphan­yl)quinoline (PQ^H^) analogue (**3**). In all three complexes, the phosphanyl­quinoline moiety acts as a bidentate *P,N*-donating chelate ligand. In the PQ^H^ complex (**3**), the Pd^II^ center has a typical planar coordination environment; however, both the methyl- and phenyl-substituted phosphanyl­quinoline (PQ^Me^ and PQ^Ph^, respectively) complexes (**1**) and (**2**) exhibit a considerable tetra­hedral distortion around the Pd^II^ center, as parameterized by the τ_4_ values of 0.1555 (4) and 0.1438 (4) for (**1**) and (**2**), respectively. The steric inter­action from the substituted group introduced at the 2-position of the quinoline ring enforces the *cis*-positioned Cl ligand to be displaced from the ideal coordination plane. Also, the ideally planar phosphanyl­quinoline five-membered chelate ring shows a large bending deformation by the displacement of the Pd^II^ center from the quinoline plane. In addition, in the phenyl-substituted complex (**3**), the coordinating quinolyl and the substituted phenyl rings are not co-planar to each other, having a dihedral angle of 33.08 (7)°. This twist conformation prohibits any inter­molecular π–π stacking inter­action between the quinoline planes, which is observed in the crystals of complexes (**1**) and (**2**).

## Abstract 2

The steric requirement resulting from the substituted methyl or phenyl group at the *ortho*-position of the coordinating quinoline-N atom in 8-(di­phenyl­phosphan­yl)quinoline enforces the square-planar coordination geometry of the di­chlorido­palladium(II) center to be highly distorted.

## Chemical context   

8-Quinolylphosphanes are competent ligands for various functional coordination compounds, because they consist of a strongly σ-donating phosphane donor group and a π-conjugated quinoline moiety and form a stable planar five-membered chelate ring on coordination to a metal center (Cai *et al.*, 2018 ▸; Hopkins *et al.*, 2019 ▸; Scattolin *et al.*, 2017 ▸). In one of our previous studies, it was revealed that 8-(di­phenyl­phosphan­yl)quinoline (PQ^H^) in the simplest di­chlorido­palladium(II) complex, [PdCl_2_(PQ^H^)] (**3**), exhibits a strong *trans* influence of the di­phenyl­phosphanyl donor group and an inter­molecular π–π stacking inter­action between the quinoline ring systems (Suzuki *et al.*, 2015 ▸). Also, in the bis­(PQ^H^)-type Ni^II^, Pd^II^ and Pt^II^ (*M* ^II^) complexes, [*M* ^II^(PQ^H^)_2_]*X*_2_ (*X* = ClO_4_, BF_4_ or CF_3_SO_3_), the *cis*(*P*,*P*)-isomers are preferably formed due to the above-mentioned *trans* influence, but the mutually *cis*-positioned quinoline groups give a steric congestion between them, causing the coordination environment around *M* ^II^ to be distorted (Suzuki, 2004 ▸; Mori *et al.*, 2020 ▸). When a methyl or phenyl group substituted at the *ortho*-position of the quinoline-N atom of PQ^H^ is used for complexation, for example in 2-methyl-8-(di­phenyl­phos­phan­yl)quinoline (PQ^Me^) (**1**) or 2-phenyl-8-(di­phenyl­phos­phan­yl)quinoline (PQ^Ph^) (**2**), a much larger steric hindrance would be expected at the *cis*-position of the coordinating quinoline-N donor site. In the present study we reveal the characteristic structural features of the simplest PdCl_2_ complexes bearing PQ^Me^ (**1**) and PQ^Ph^ (**2**) chelate ligands.
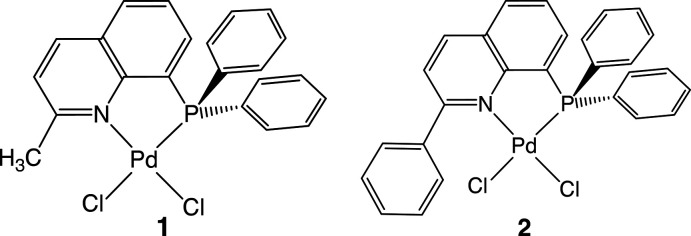


## Structural commentary   

In the crystals of (**1**) and (**2**), the quinolylphosphane moiety coordinates to a Pd^II^ center in a bidentate κ^2^ *P,N* mode, and the coordination environment around the Pd^II^ center is roughly square-planar with two additional chlorido ligands. The analogous PQ^H^ complex (**3**) has a typical planar environment (Suzuki *et al.*, 2015 ▸); the *τ*_4_ value (Yang *et al.*, 2007 ▸) indicating the tetra­hedral distortion around the four-coordinate Pd^II^ center is here only 0.0552 (4). The coordination plane (defined by the central Pd and four donor atoms) and the quinoline plane are almost co-planar, with their dihedral angle being 8.58 (3)°. The Pd1—P1 and Pd1—N1 bond lengths in the structure of (**3**) are 2.2026 (6) and 2.065 (2) Å, respectively, and the P1—Pd1—N1 chelate bite angle is 84.75 (6)°. The Pd—Cl1 (*trans* to P1) and Pd1—Cl2 (*trans* to N1) bond lengths are 2.3716 (7) and 2.2885 (8) Å, respectively, indicating a strong *trans* influence of the Ph_2_P– donor group.

**Figure 1:**
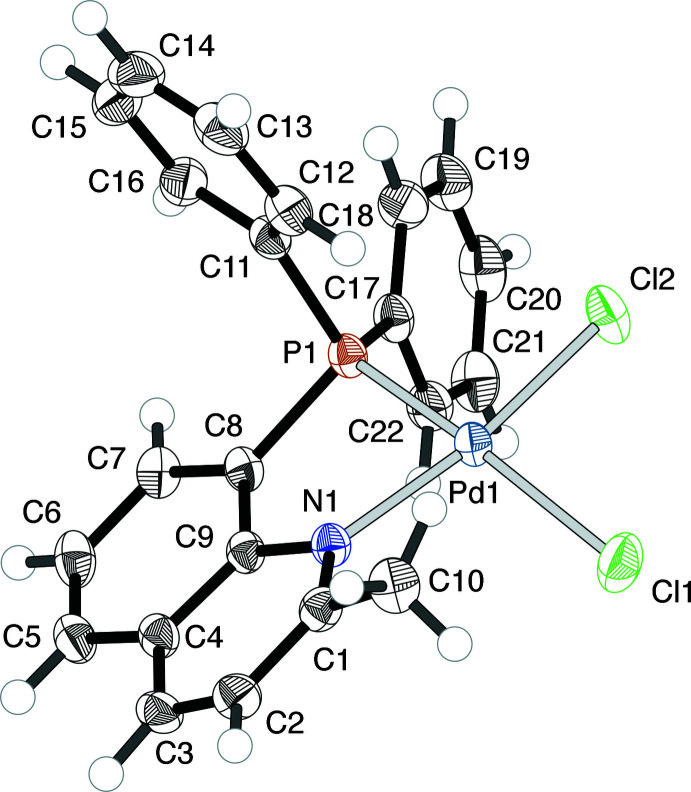
The mol­ecular structure of [PdCl_2_(PQ^Me^)] (**1**), showing the atom-numbering scheme and displacement ellipsoids at the 50% probability level.

**Figure 2:**
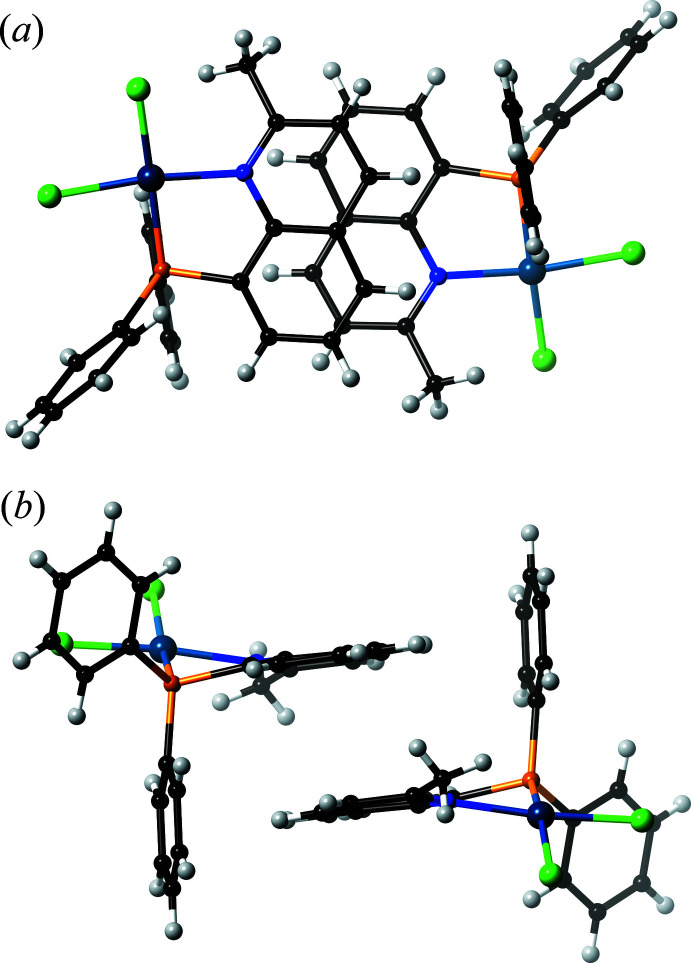
Perspective (*a*) top and (*b*) side views of a dimeric unit of (**1**), showing the distortion of the coordination environment around Pd^II^ and the inter­molecular π–π stacking inter­action between the quinoline ring systems. Color code: Pd, blueish purple; P, orange; N, blue; C, black and H, gray.

In the PQ^Me^ complex (**1**) (Fig. 1 ▸), the coordination environment around the Pd^II^ center is apparently distorted; the τ_4_ value is 0.1555 (4). The steric requirement from the 2-methyl substituent of the coordinating quinoline group causes the Cl ligand in the *cis*-position to be pushed away (Fig. 2 ▸). Thus, the Cl1 atom is considerably displaced from the Pd^II^ coordination plane (defined by Pd1, Cl2, P1 and N1) by 0.554 (1) Å, and the P1—Pd1—Cl1 bond angle is 166.74 (2)°, as compared to the N1—Pd1—Cl2 angle of 171.32 (5)°. More importantly, the PQ^Me^ chelate ring is no longer planar. The Pd1 atom is displaced by 0.755 (2) Å from the chelating ligand plane (defined by P1, C8, C9 and N1), and the dihedral angle *φ*_C_ between the plane [Pd1,P1,N1] and the quinoline plane (defined by N1 and C1–C9) is 25.35 (3)°. Thus, an envelope-type deformation of the chelate ring is observed. This distortion would weaken the Pd—N bond, because the direction of the lone-pair electrons on the N atom does not match with the Pd^II^ acceptor *d*-orbital. In fact, the Pd1—N1 bond length of 2.0971 (17) Å in (**1**) is slightly longer than that in (**3**). Other coordination bonds and angles are collated in Table 1 ▸ and are comparable to those in (**3**).

**Figure 3:**
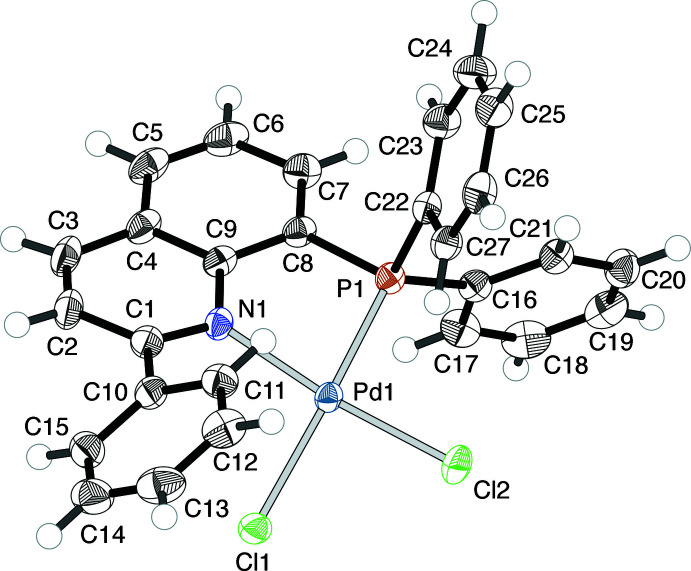
The mol­ecular structure of [PdCl_2_(PQ^Ph^)] (**2**), showing the atom-numbering scheme and displacement ellipsoids at the 50% probability level.

The PQ^Ph^ complex (**2**) shows a more explicit distortion of the coordination environment on the quinolylphosphane ligand due to the 2-phenyl substitution group (Figs. 3 ▸ and 4 ▸). The τ_4_ value is 0.1438 (4), and the Cl1 atom is displaced from the Pd^II^ coordination plane (defined by Pd1, Cl2, P1 and N1) by 0.571 (1) Å. The P1—Pd1—Cl1 and N1—Pd1—Cl2 bond angles are 165.930 (19) and 173.78 (5)°, respectively. The Pd1 atom is displaced by 0.864 (2) Å from the chelating ligand plane (defined by P1, C8, C9 and N1), and the dihedral angle *φ*_C_ between the plane [Pd1,P1,N1] and the quinoline plane (defined by N1 and C1–C9) is 32.56 (3)°. In addition, the substituted phenyl plane is twisted from the attached quinoline plane with a dihedral angle of 33.08 (7)°. Table 2 ▸ lists selected bond lengths and angles.

## Supra­molecular features   

**Figure 4:**
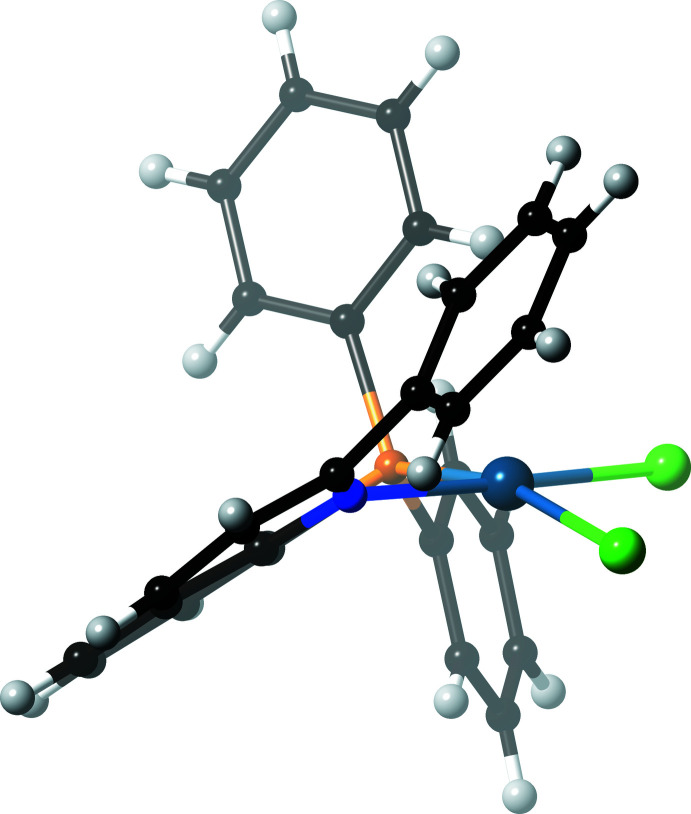
A perspective side view of (**2**), showing the distortion of the coordination environment around Pd^II^. Color code: Pd, blueish purple; P, orange; N, blue; C, black and H, gray.

**Figure 5:**
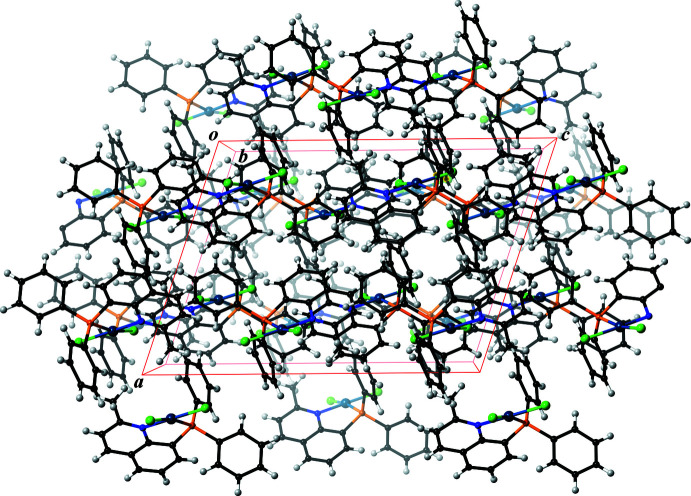
The packing of [PdCl_2_(PQ^Me^)] (**1**), viewed along the *b* axis. Color code: Pd, blueish purple; P, orange; N, blue; C, black and H, gray.

In the crystal structure, the mol­ecular PQ^H^ complex (**3**) forms a dimer by an inter­molecular π–π stacking inter­action between the quinoline ring systems. A similar stacking inter­action is observed in the crystal structure of the PQ^Me^ complex (**1**) (Fig. 2 ▸). The shortest inter­molecular contact distance is 3.322 (3) Å for C2⋯C6^i^ [symmetry code: (i) −*x* + 1, −*y* + 1, −*z*). By contrast, the PQ^Ph^ complex (**2**) does not show a similar stacking inter­action to the above examples, because the twist motion of the attached phenyl group prohibits a full π–π stacking inter­action between the mol­ecules (Fig. 4 ▸). The shortest inter­molecular contact distance in (**2**) is 3.549 (3) Å for C2⋯C3^ii^ [symmetry code (ii) −*x* + 1, −*y* + 2, −*z* + 1]. There are no other obvious supra­molecular features in the crystal structures of (**1**) and (**2**) (Figs. 5 ▸ and 6 ▸).

## Database survey   

Crystal structures of the following transition-metal complexes containing PQ^Me^ or PQ^Ph^ were retrieved from the Cambridge Structural Database (CSD, version 5.41, last update May 2020; Groom *et al.*, 2016 ▸): [Cu(PQ^Me^)_2_]PF_6_ (refcode NOPNOW; Tsukuda *et al.*, 2009 ▸), [Cu(PQ^Me^){(Ph_2_PC_6_H_4_)_2_O}]BF_4_·CH_2_Cl_2_ (OGUYEV; Qin *et al.*, 2009 ▸), [{Ni(PQ^Me^)Cl}_2_(μ-Cl)_2_]·CH_2_Cl_2_ (MUMDAZ; Sun *et al.*, 2002 ▸), two organometallic Pd^II^ complexes (BUPMIK and BUPMOQ; Canovese *et al.*, 2015 ▸). We have recently reported some Ni^II^, Pd^II^ and Pt^II^ (*M* ^II^) complexes bearing PQ^R^: [*M*(PQ^R^)_2_]*X*_2_ (*X* = Br, BF_4_ or CF_3_SO_3_) (Mori *et al.*, 2020 ▸) and [Pt(ppy)(PQ^R^)]BF_4_ [ppy = 2-(2′-pyrid­yl)phenyl; Mori & Suzuki, 2020 ▸]. A related palladium(II) complex containing 2-methyl-8-(methyl­phenyl­phosphan­yl)quinoline has also been reported (PUMDAD; Bock *et al.*, 2010 ▸).

## Synthesis and crystallization   

The ligands, PQ^Me^ and PQ^Ph^, were prepared according to the methods reported previously (Mori & Suzuki, 2020 ▸). Complex (**1**) was prepared as follows: under a nitro­gen atmosphere, a di­chloro­methane solution (10 ml) of PQ^Me^ (0.109 g, 0.334 mmol) was added under stirring to a di­chloro­methane solution (8 ml) of [PdCl_2_(PhCN)_2_] (PhCN = benzo­nitrile) (0.121 g, 0.315 mmol), and the mixture was stirred overnight at room temperature. The resulting solution was concentrated using a rotary evaporator, and diethyl ether was added under stirring to the concentrate, giving a yellow precipitate, which was collected by filtration, washed with diethyl ether (10 ml), and dried *in vacuo*. Yield: 0.142 g (92%). Analysis found: C, 51.03; H, 3.67; N, 2.99%. Calculated for C_22_H_18_Cl_2_NPPd·0.7H_2_O: C, 51.08; H, 3.78; N, 2.71%. Yellow needle-like crystals of (**1**) were obtained by recrystallization from an aceto­nitrile solution by diffusion of diisopropyl ether.

Complex (**2**) was prepared as follows: under a nitro­gen atmosphere, a di­chloro­methane solution (10 ml) of PQ^Ph^ (0.071 g, 0.18 mmol) was added under stirring to a di­chloro­methane solution (10 ml) of [PdCl_2_(PhCN)_2_] (0.070 g, 0.18 mmol), and the mixture was stirred overnight at room temperature. The resulting brown precipitate was filtered off, and the filtrate was concentrated under reduced pressure. Diethyl ether was added under stirring to the concentrate, giving a yellow precipitate, which was collected by filtration, washed with diethyl ether (10 ml), and dried *in vacuo*. Yield: 0.041 g (40%). Analysis found: C, 56.23; H, 3.50; N, 2.56%. Calculated for C_27_H_20_Cl_2_NPPd: C, 57.22; H, 3.56; N, 2.47%. Yellow block-like crystals of (**2**) were obtained by recrystallization from an aceto­nitrile solution by diffusion of diisopropyl ether.

## Refinement   

Crystal data, data collection and structure refinement details are summarized in Table 3 ▸. All H atoms were positioned geometrically and refined using a riding model, with C—H = 0.95 (aromatic) or 0.98 (meth­yl) Å and *U*_iso_ = 1.2*U*_eq_(C). For the refinement of (**2**), two reflections (4
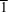
1, 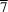
98) were omitted because they showed a significantly lower intensity than calculated, most probably caused by obstruction from the beam stop.

## Supplementary Material

Crystal structure: contains datablock(s) complex1, complex2. DOI: 10.1107/S2056989020016096/wm5591sup1.cif

Structure factors: contains datablock(s) complex1. DOI: 10.1107/S2056989020016096/wm5591complex1sup4.hkl

Structure factors: contains datablock(s) complex2. DOI: 10.1107/S2056989020016096/wm5591complex2sup5.hkl

CCDC references: 2049480, 2049479

Additional supporting information:  crystallographic information; 3D view; checkCIF report

## Figures and Tables

**Figure 6:**
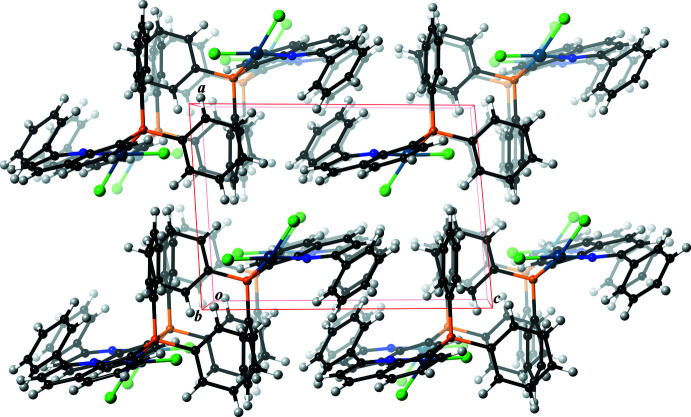
The packing of [PdCl_2_(PQ^Ph^)] (**2**), viewed along the *b* axis. Color code: Pd, blueish purple; P, orange; N, blue; C, black and H, gray.

**Table 1:** Selected geometric parameters (Å, °) for complex (**1**)

Pd1—N1	2.0971 (17)	Pd1—Cl2	2.2823 (6)
Pd1—P1	2.1910 (5)	Pd1—Cl1	2.3742 (6)
			
N1—Pd1—P1	83.52 (5)	N1—Pd1—Cl1	98.30 (5)
N1—Pd1—Cl2	171.32 (5)	P1—Pd1—Cl1	166.74 (2)
P1—Pd1—Cl2	88.25 (2)	Cl2—Pd1—Cl1	90.34 (2)

**Table 2:** Selected geometric parameters (Å, °) for complex (**2**)

Pd1—N1	2.0806 (15)	Pd1—Cl2	2.2769 (5)
Pd1—P1	2.2036 (6)	Pd1—Cl1	2.3738 (6)
			
N1—Pd1—P1	83.24 (5)	N1—Pd1—Cl1	94.56 (5)
N1—Pd1—Cl2	173.78 (5)	P1—Pd1—Cl1	165.930 (19)
P1—Pd1—Cl2	90.56 (2)	Cl2—Pd1—Cl1	91.57 (2)

**Table 3:** Experimental details

	Complex (**1**)	Complex (**2**)
Crystal data		
Chemical formula	[PdCl_2_(C_22_H_18_NP)]	[PdCl_2_(C_27_H_20_NP)]
*M*_r_	504.64	566.71
Crystal system, space group	Monoclinic, *C*2/*c*	Triclinic, *P* 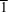
Temperature (K)	188	188
*a*, *b*, *c* (Å)	13.8153 (5), 15.5676 (8), 18.9683 (5)	9.6582 (13), 9.8765 (14), 13.0748 (14)
α, β, γ (°)	90, 108.733 (2), 90	102.011 (4), 90.426 (4), 109.827 (4)
*V* (Å^3^)	3863.4 (3)	1143.4 (3)
*Z*	8	2
Radiation type	Mo *K*α	Mo *K*α
μ (mm^−1^)	1.33	1.13
Crystal size (mm)	0.50 × 0.20 × 0.15	0.30 × 0.20 × 0.20

Data collection		
Diffractometer	Rigaku R-AXIS RAPID	Rigaku R-AXIS RAPID
Absorption correction	Numerical (*NUMABS*; Rigaku, 1999 ▸)	Numerical (*NUMABS*; Rigaku, 1999 ▸)
*T*_min_, *T*_max_	0.662, 0.819	0.640, 0.797
No. of measured, independent and observed [*I* > 2σ(*I*)] reflections	18561, 4438, 3976	11339, 5194, 4682
*R*_int_	0.039	0.027
(sin θ/λ)_max_ (Å^−1^)	0.649	0.649

Refinement		
*R*[*F* ^2^ > 2σ(*F* ^2^)], *wR*(*F* ^2^), *S*	0.025, 0.063, 1.05	0.025, 0.064, 1.06
No. of reflections	4438	5192
No. of parameters	245	289
H-atom treatment	H-atom parameters constrained	H-atom parameters constrained
Δρ_max_, Δρ_min_ (e Å^−3^)	0.46, −0.34	0.74, −0.59

Computer programs: *PROCESS-AUTO* (Rigaku, 1998 ▸), *CrystalStructure* (Rigaku, 2010 ▸), *DIRDIF99* (Beurskens *et al.*, 1999 ▸), *Il Milione* (Burla *et al.*, 2012 ▸), *SHELXL2014/7* (Sheldrick, 2015 ▸), *CrystalMaker* (CrystalMaker, 2017 ▸), and *publCIF* (Westrip, 2010 ▸).

## supplementary crystallographic information

### Dichlorido[8-(diphenylphosphanyl)-2-methylquinoline-κ2N,P]palladium(II) (complex1) Crystal data

**Table:**

[PdCl_2_(C_22_H_18_NP)]	*F*(000) = 2016
*M**_r_* = 504.64	*D*_x_ = 1.735 Mg m^−^^3^
Monoclinic, *C*2/*c*	Mo *K*α radiation, λ = 0.71075 Å
*a* = 13.8153 (5) Å	Cell parameters from 15062 reflections
*b* = 15.5676 (8) Å	θ = 3.1–27.6°
*c* = 18.9683 (5) Å	µ = 1.33 mm^−^^1^
β = 108.733 (2)°	*T* = 188 K
*V* = 3863.4 (3) Å^3^	Needle, yellow
*Z* = 8	0.50 × 0.20 × 0.15 mm

### Dichlorido[8-(diphenylphosphanyl)-2-methylquinoline-κ2N,P]palladium(II) (complex1) Data collection

**Table:**

Rigaku R-AXIS RAPID diffractometer	4438 independent reflections
Radiation source: fine-focus sealed tube	3976 reflections with *I* > 2σ(*I*)
Detector resolution: 10.000 pixels mm^-1^	*R*_int_ = 0.039
ω scans	θ_max_ = 27.5°, θ_min_ = 3.1°
Absorption correction: numerical (NUMABS; Rigaku, 1999)	*h* = −17→17
*T*_min_ = 0.662, *T*_max_ = 0.819	*k* = −20→20
18561 measured reflections	*l* = −23→25

### Dichlorido[8-(diphenylphosphanyl)-2-methylquinoline-κ2N,P]palladium(II) (complex1) Refinement

**Table:**

Refinement on *F*^2^	Primary atom site location: structure-invariant direct methods
Least-squares matrix: full	Secondary atom site location: difference Fourier map
*R*[*F*^2^ > 2σ(*F*^2^)] = 0.025	Hydrogen site location: inferred from neighbouring sites
*wR*(*F*^2^) = 0.063	H-atom parameters constrained
*S* = 1.05	*w* = 1/[σ^2^(*F*_o_^2^) + (0.0257*P*)^2^ + 5.1054*P*] where *P* = (*F*_o_^2^ + 2*F*_c_^2^)/3
4438 reflections	(Δ/σ)_max_ < 0.001
245 parameters	Δρ_max_ = 0.46 e Å^−^^3^
0 restraints	Δρ_min_ = −0.34 e Å^−^^3^

### Dichlorido[8-(diphenylphosphanyl)-2-methylquinoline-κ2N,P]palladium(II) (complex1) Special details

**Table:**

Geometry. All esds (except the esd in the dihedral angle between two l.s. planes) are estimated using the full covariance matrix. The cell esds are taken into account individually in the estimation of esds in distances, angles and torsion angles; correlations between esds in cell parameters are only used when they are defined by crystal symmetry. An approximate (isotropic) treatment of cell esds is used for estimating esds involving l.s. planes.

### Dichlorido[8-(diphenylphosphanyl)-2-methylquinoline-κ2N,P]palladium(II) (complex1) Fractional atomic coordinates and isotropic or equivalent isotropic displacement parameters (Å^2^)

**Table:**

	*x*	*y*	*z*	*U*_iso_*/*U*_eq_	
Pd1	0.18958 (2)	0.00853 (2)	0.12906 (2)	0.02335 (6)	
Cl1	0.18633 (5)	−0.13050 (4)	0.07691 (4)	0.04342 (15)	
Cl2	0.14113 (5)	−0.04783 (4)	0.22366 (3)	0.03709 (14)	
P1	0.22803 (4)	0.12790 (3)	0.19320 (3)	0.02356 (11)	
N1	0.23191 (12)	0.07847 (11)	0.04910 (9)	0.0238 (3)	
C1	0.20531 (16)	0.06113 (14)	−0.02343 (12)	0.0274 (4)	
C2	0.26023 (17)	0.09721 (15)	−0.06751 (12)	0.0314 (5)	
H2	0.2439	0.0806	−0.1182	0.038*	
C3	0.33556 (16)	0.15490 (15)	−0.03855 (12)	0.0308 (5)	
H3	0.3744	0.1764	−0.0679	0.037*	
C4	0.35637 (15)	0.18305 (14)	0.03552 (12)	0.0269 (4)	
C5	0.42756 (16)	0.24877 (15)	0.06901 (13)	0.0314 (5)	
H5	0.4667	0.2742	0.0415	0.038*	
C6	0.44088 (16)	0.27612 (15)	0.13956 (13)	0.0334 (5)	
H6	0.4893	0.3199	0.1610	0.040*	
C7	0.38300 (16)	0.23959 (14)	0.18060 (12)	0.0303 (5)	
H7	0.3902	0.2604	0.2291	0.036*	
C8	0.31559 (15)	0.17359 (13)	0.15078 (11)	0.0249 (4)	
C9	0.30212 (14)	0.14371 (13)	0.07806 (11)	0.0232 (4)	
C10	0.11417 (19)	0.00666 (15)	−0.06008 (13)	0.0354 (5)	
H10A	0.0587	0.0218	−0.0406	0.042*	
H10B	0.1321	−0.0540	−0.0497	0.042*	
H10C	0.0919	0.0164	−0.1140	0.042*	
C11	0.12787 (16)	0.20610 (14)	0.17993 (12)	0.0268 (4)	
C12	0.03231 (17)	0.18947 (16)	0.12721 (13)	0.0340 (5)	
H12	0.0190	0.1349	0.1037	0.041*	
C13	−0.04303 (18)	0.25209 (18)	0.10918 (15)	0.0405 (6)	
H13	−0.1078	0.2408	0.0733	0.049*	
C14	−0.0230 (2)	0.33035 (18)	0.14365 (15)	0.0447 (6)	
H14	−0.0744	0.3735	0.1318	0.054*	
C15	0.0717 (2)	0.34742 (17)	0.19588 (15)	0.0425 (6)	
H15	0.0843	0.4023	0.2190	0.051*	
C16	0.14790 (18)	0.28583 (15)	0.21475 (13)	0.0338 (5)	
H16	0.2124	0.2977	0.2506	0.041*	
C17	0.29830 (16)	0.11805 (14)	0.29131 (11)	0.0270 (4)	
C18	0.25801 (18)	0.14295 (16)	0.34621 (13)	0.0349 (5)	
H18	0.1928	0.1697	0.3333	0.042*	
C19	0.3144 (2)	0.12828 (17)	0.42099 (13)	0.0404 (6)	
H19	0.2880	0.1471	0.4589	0.048*	
C20	0.4061 (2)	0.08758 (16)	0.44024 (13)	0.0402 (6)	
H20	0.4427	0.0771	0.4912	0.048*	
C21	0.44627 (18)	0.06140 (16)	0.38595 (13)	0.0384 (5)	
H21	0.5102	0.0325	0.3994	0.046*	
C22	0.39295 (17)	0.07739 (15)	0.31183 (13)	0.0325 (5)	
H22	0.4213	0.0604	0.2745	0.039*	

### Dichlorido[8-(diphenylphosphanyl)-2-methylquinoline-κ2N,P]palladium(II) (complex1) Atomic displacement parameters (Å^2^)

**Table:**

	*U*^11^	*U*^22^	*U*^33^	*U*^12^	*U*^13^	*U*^23^
Pd1	0.02644 (9)	0.02137 (9)	0.02156 (9)	−0.00327 (6)	0.00677 (6)	0.00066 (6)
Cl1	0.0661 (4)	0.0244 (3)	0.0403 (3)	−0.0008 (3)	0.0178 (3)	−0.0036 (2)
Cl2	0.0465 (3)	0.0371 (3)	0.0281 (3)	−0.0135 (2)	0.0126 (2)	0.0043 (2)
P1	0.0270 (3)	0.0234 (3)	0.0196 (2)	−0.0033 (2)	0.00651 (19)	0.00029 (19)
N1	0.0253 (8)	0.0235 (8)	0.0218 (8)	−0.0011 (7)	0.0062 (6)	0.0011 (7)
C1	0.0313 (10)	0.0252 (10)	0.0247 (10)	0.0031 (8)	0.0075 (8)	0.0007 (8)
C2	0.0386 (12)	0.0347 (12)	0.0215 (10)	0.0043 (10)	0.0103 (9)	0.0026 (9)
C3	0.0327 (11)	0.0361 (12)	0.0273 (11)	0.0065 (9)	0.0148 (9)	0.0089 (9)
C4	0.0256 (10)	0.0276 (10)	0.0279 (11)	0.0037 (8)	0.0089 (8)	0.0078 (8)
C5	0.0249 (10)	0.0320 (11)	0.0377 (12)	−0.0006 (9)	0.0106 (9)	0.0096 (9)
C6	0.0271 (10)	0.0286 (11)	0.0394 (13)	−0.0060 (9)	0.0034 (9)	0.0062 (9)
C7	0.0299 (10)	0.0285 (11)	0.0285 (11)	−0.0026 (9)	0.0037 (8)	0.0009 (9)
C8	0.0242 (10)	0.0249 (10)	0.0244 (10)	−0.0014 (8)	0.0061 (8)	0.0037 (8)
C9	0.0228 (9)	0.0223 (9)	0.0236 (10)	0.0011 (8)	0.0060 (7)	0.0038 (8)
C10	0.0394 (13)	0.0378 (13)	0.0252 (12)	−0.0055 (10)	0.0049 (9)	−0.0021 (9)
C11	0.0292 (10)	0.0283 (10)	0.0247 (10)	−0.0004 (8)	0.0110 (8)	0.0039 (8)
C12	0.0313 (11)	0.0362 (12)	0.0348 (12)	−0.0044 (10)	0.0112 (9)	0.0020 (10)
C13	0.0300 (11)	0.0514 (16)	0.0405 (14)	0.0025 (11)	0.0119 (10)	0.0120 (12)
C14	0.0473 (14)	0.0491 (16)	0.0450 (15)	0.0180 (12)	0.0249 (12)	0.0143 (12)
C15	0.0608 (16)	0.0334 (13)	0.0396 (14)	0.0096 (12)	0.0250 (12)	0.0037 (11)
C16	0.0408 (12)	0.0312 (12)	0.0302 (12)	0.0009 (10)	0.0128 (9)	−0.0003 (9)
C17	0.0327 (11)	0.0252 (10)	0.0217 (10)	−0.0050 (8)	0.0068 (8)	0.0008 (8)
C18	0.0418 (13)	0.0364 (12)	0.0272 (11)	0.0009 (10)	0.0122 (9)	0.0004 (10)
C19	0.0567 (15)	0.0411 (14)	0.0244 (12)	−0.0079 (12)	0.0145 (10)	−0.0019 (10)
C20	0.0528 (15)	0.0378 (13)	0.0218 (11)	−0.0103 (11)	0.0006 (10)	0.0043 (10)
C21	0.0358 (12)	0.0373 (13)	0.0347 (13)	−0.0025 (10)	0.0012 (10)	0.0072 (10)
C22	0.0351 (11)	0.0324 (12)	0.0294 (12)	−0.0014 (9)	0.0095 (9)	0.0020 (9)

### Dichlorido[8-(diphenylphosphanyl)-2-methylquinoline-κ2N,P]palladium(II) (complex1) Geometric parameters (Å, º)

**Table:**

Pd1—N1	2.0971 (17)	C10—H10B	0.9800
Pd1—P1	2.1910 (5)	C10—H10C	0.9800
Pd1—Cl2	2.2823 (6)	C11—C16	1.391 (3)
Pd1—Cl1	2.3742 (6)	C11—C12	1.401 (3)
P1—C11	1.799 (2)	C12—C13	1.386 (3)
P1—C8	1.800 (2)	C12—H12	0.9500
P1—C17	1.808 (2)	C13—C14	1.368 (4)
N1—C1	1.333 (3)	C13—H13	0.9500
N1—C9	1.390 (3)	C14—C15	1.390 (4)
C1—C2	1.413 (3)	C14—H14	0.9500
C1—C10	1.492 (3)	C15—C16	1.384 (3)
C2—C3	1.351 (3)	C15—H15	0.9500
C2—H2	0.9500	C16—H16	0.9500
C3—C4	1.410 (3)	C17—C18	1.384 (3)
C3—H3	0.9500	C17—C22	1.391 (3)
C4—C9	1.406 (3)	C18—C19	1.401 (3)
C4—C5	1.420 (3)	C18—H18	0.9500
C5—C6	1.359 (3)	C19—C20	1.358 (4)
C5—H5	0.9500	C19—H19	0.9500
C6—C7	1.403 (3)	C20—C21	1.379 (4)
C6—H6	0.9500	C20—H20	0.9500
C7—C8	1.380 (3)	C21—C22	1.384 (3)
C7—H7	0.9500	C21—H21	0.9500
C8—C9	1.410 (3)	C22—H22	0.9500
C10—H10A	0.9800		
			
N1—Pd1—P1	83.52 (5)	C1—C10—H10A	109.5
N1—Pd1—Cl2	171.32 (5)	C1—C10—H10B	109.5
P1—Pd1—Cl2	88.25 (2)	H10A—C10—H10B	109.5
N1—Pd1—Cl1	98.30 (5)	C1—C10—H10C	109.5
P1—Pd1—Cl1	166.74 (2)	H10A—C10—H10C	109.5
Cl2—Pd1—Cl1	90.34 (2)	H10B—C10—H10C	109.5
C11—P1—C8	105.50 (10)	C16—C11—C12	120.1 (2)
C11—P1—C17	109.64 (10)	C16—C11—P1	120.69 (17)
C8—P1—C17	106.13 (10)	C12—C11—P1	118.75 (17)
C11—P1—Pd1	117.25 (7)	C13—C12—C11	120.5 (2)
C8—P1—Pd1	99.48 (7)	C13—C12—H12	119.8
C17—P1—Pd1	116.99 (7)	C11—C12—H12	119.8
C1—N1—C9	118.82 (18)	C14—C13—C12	119.2 (2)
C1—N1—Pd1	126.37 (14)	C14—C13—H13	120.4
C9—N1—Pd1	114.50 (13)	C12—C13—H13	120.4
N1—C1—C2	120.5 (2)	C13—C14—C15	120.7 (2)
N1—C1—C10	120.52 (19)	C13—C14—H14	119.7
C2—C1—C10	118.9 (2)	C15—C14—H14	119.7
C3—C2—C1	120.9 (2)	C16—C15—C14	121.1 (2)
C3—C2—H2	119.6	C16—C15—H15	119.5
C1—C2—H2	119.6	C14—C15—H15	119.5
C2—C3—C4	119.7 (2)	C15—C16—C11	118.5 (2)
C2—C3—H3	120.1	C15—C16—H16	120.8
C4—C3—H3	120.1	C11—C16—H16	120.8
C9—C4—C3	117.4 (2)	C18—C17—C22	119.1 (2)
C9—C4—C5	118.5 (2)	C18—C17—P1	122.53 (17)
C3—C4—C5	124.0 (2)	C22—C17—P1	118.19 (16)
C6—C5—C4	121.4 (2)	C17—C18—C19	119.3 (2)
C6—C5—H5	119.3	C17—C18—H18	120.4
C4—C5—H5	119.3	C19—C18—H18	120.4
C5—C6—C7	119.9 (2)	C20—C19—C18	121.1 (2)
C5—C6—H6	120.1	C20—C19—H19	119.5
C7—C6—H6	120.1	C18—C19—H19	119.5
C8—C7—C6	120.3 (2)	C19—C20—C21	120.1 (2)
C8—C7—H7	119.9	C19—C20—H20	119.9
C6—C7—H7	119.9	C21—C20—H20	119.9
C7—C8—C9	120.40 (19)	C20—C21—C22	119.7 (2)
C7—C8—P1	124.94 (17)	C20—C21—H21	120.2
C9—C8—P1	114.22 (15)	C22—C21—H21	120.2
N1—C9—C4	121.63 (19)	C21—C22—C17	120.8 (2)
N1—C9—C8	118.93 (18)	C21—C22—H22	119.6
C4—C9—C8	119.37 (19)	C17—C22—H22	119.6
			
C9—N1—C1—C2	11.2 (3)	C7—C8—C9—C4	−1.4 (3)
Pd1—N1—C1—C2	−162.19 (16)	P1—C8—C9—C4	171.46 (15)
C9—N1—C1—C10	−165.95 (19)	C8—P1—C11—C16	−66.1 (2)
Pd1—N1—C1—C10	20.7 (3)	C17—P1—C11—C16	47.8 (2)
N1—C1—C2—C3	−5.1 (3)	Pd1—P1—C11—C16	−175.63 (15)
C10—C1—C2—C3	172.1 (2)	C8—P1—C11—C12	106.50 (18)
C1—C2—C3—C4	−3.3 (3)	C17—P1—C11—C12	−139.61 (17)
C2—C3—C4—C9	5.1 (3)	Pd1—P1—C11—C12	−3.1 (2)
C2—C3—C4—C5	−174.3 (2)	C16—C11—C12—C13	0.1 (3)
C9—C4—C5—C6	−2.6 (3)	P1—C11—C12—C13	−172.52 (18)
C3—C4—C5—C6	176.8 (2)	C11—C12—C13—C14	−0.2 (4)
C4—C5—C6—C7	−0.5 (3)	C12—C13—C14—C15	0.3 (4)
C5—C6—C7—C8	2.6 (3)	C13—C14—C15—C16	−0.4 (4)
C6—C7—C8—C9	−1.7 (3)	C14—C15—C16—C11	0.3 (4)
C6—C7—C8—P1	−173.74 (17)	C12—C11—C16—C15	−0.1 (3)
C11—P1—C8—C7	74.4 (2)	P1—C11—C16—C15	172.32 (18)
C17—P1—C8—C7	−41.9 (2)	C11—P1—C17—C18	20.9 (2)
Pd1—P1—C8—C7	−163.70 (17)	C8—P1—C17—C18	134.36 (19)
C11—P1—C8—C9	−98.03 (17)	Pd1—P1—C17—C18	−115.78 (18)
C17—P1—C8—C9	145.66 (15)	C11—P1—C17—C22	−164.51 (17)
Pd1—P1—C8—C9	23.84 (16)	C8—P1—C17—C22	−51.0 (2)
C1—N1—C9—C4	−9.3 (3)	Pd1—P1—C17—C22	58.83 (19)
Pd1—N1—C9—C4	164.82 (15)	C22—C17—C18—C19	1.3 (3)
C1—N1—C9—C8	167.77 (19)	P1—C17—C18—C19	175.92 (18)
Pd1—N1—C9—C8	−18.1 (2)	C17—C18—C19—C20	−2.2 (4)
C3—C4—C9—N1	1.1 (3)	C18—C19—C20—C21	1.3 (4)
C5—C4—C9—N1	−179.45 (18)	C19—C20—C21—C22	0.5 (4)
C3—C4—C9—C8	−175.94 (19)	C20—C21—C22—C17	−1.3 (4)
C5—C4—C9—C8	3.5 (3)	C18—C17—C22—C21	0.4 (3)
C7—C8—C9—N1	−178.54 (19)	P1—C17—C22—C21	−174.44 (18)
P1—C8—C9—N1	−5.7 (2)		

### Dichlorido[8-(diphenylphosphanyl)-2-phenylquinoline-κ2N</i.,P]palladium(II) (complex2) Crystal data

**Table:**

[PdCl_2_(C_27_H_20_NP)]	*Z* = 2
*M**_r_* = 566.71	*F*(000) = 568
Triclinic, *P*1	*D*_x_ = 1.646 Mg m^−^^3^
*a* = 9.6582 (13) Å	Mo *K*α radiation, λ = 0.71075 Å
*b* = 9.8765 (14) Å	Cell parameters from 9246 reflections
*c* = 13.0748 (14) Å	θ = 3.0–27.5°
α = 102.011 (4)°	µ = 1.13 mm^−^^1^
β = 90.426 (4)°	*T* = 188 K
γ = 109.827 (4)°	Block, yellow
*V* = 1143.4 (3) Å^3^	0.30 × 0.20 × 0.20 mm

### Dichlorido[8-(diphenylphosphanyl)-2-phenylquinoline-κ2N</i.,P]palladium(II) (complex2) Data collection

**Table:**

Rigaku R-AXIS RAPID diffractometer	5194 independent reflections
Radiation source: fine-focus sealed tube	4682 reflections with *I* > 2σ(*I*)
Detector resolution: 10.000 pixels mm^-1^	*R*_int_ = 0.027
ω scans	θ_max_ = 27.5°, θ_min_ = 3.0°
Absorption correction: numerical (NUMABS; Rigaku, 1999)	*h* = −10→12
*T*_min_ = 0.640, *T*_max_ = 0.797	*k* = −12→12
11339 measured reflections	*l* = −16→16

### Dichlorido[8-(diphenylphosphanyl)-2-phenylquinoline-κ2N</i.,P]palladium(II) (complex2) Refinement

**Table:**

Refinement on *F*^2^	Primary atom site location: structure-invariant direct methods
Least-squares matrix: full	Secondary atom site location: difference Fourier map
*R*[*F*^2^ > 2σ(*F*^2^)] = 0.025	Hydrogen site location: inferred from neighbouring sites
*wR*(*F*^2^) = 0.064	H-atom parameters constrained
*S* = 1.06	*w* = 1/[σ^2^(*F*_o_^2^) + (0.0323*P*)^2^ + 0.2021*P*] where *P* = (*F*_o_^2^ + 2*F*_c_^2^)/3
5192 reflections	(Δ/σ)_max_ = 0.001
289 parameters	Δρ_max_ = 0.74 e Å^−^^3^
0 restraints	Δρ_min_ = −0.59 e Å^−^^3^

### Dichlorido[8-(diphenylphosphanyl)-2-phenylquinoline-κ2N</i.,P]palladium(II) (complex2) Special details

**Table:**

Geometry. All esds (except the esd in the dihedral angle between two l.s. planes) are estimated using the full covariance matrix. The cell esds are taken into account individually in the estimation of esds in distances, angles and torsion angles; correlations between esds in cell parameters are only used when they are defined by crystal symmetry. An approximate (isotropic) treatment of cell esds is used for estimating esds involving l.s. planes.

### Dichlorido[8-(diphenylphosphanyl)-2-phenylquinoline-κ2N</i.,P]palladium(II) (complex2) Fractional atomic coordinates and isotropic or equivalent isotropic displacement parameters (Å^2^)

**Table:**

	*x*	*y*	*z*	*U*_iso_*/*U*_eq_	
Pd1	0.25082 (2)	1.07058 (2)	0.24836 (2)	0.02194 (6)	
Cl1	0.43029 (5)	1.29486 (5)	0.34148 (4)	0.02876 (11)	
Cl2	0.24428 (6)	1.16677 (6)	0.10529 (4)	0.03346 (12)	
P1	0.13193 (5)	0.84625 (6)	0.15446 (4)	0.02330 (11)	
N1	0.24296 (17)	0.96063 (18)	0.36894 (12)	0.0247 (3)	
C1	0.2588 (2)	1.0232 (2)	0.47079 (15)	0.0286 (4)	
C2	0.3092 (2)	0.9587 (3)	0.54451 (16)	0.0352 (5)	
H2	0.3254	1.0066	0.6168	0.042*	
C3	0.3343 (2)	0.8301 (3)	0.51319 (16)	0.0358 (5)	
H3	0.3758	0.7927	0.5622	0.043*	
C4	0.2985 (2)	0.7512 (2)	0.40669 (16)	0.0307 (4)	
C5	0.3080 (2)	0.6111 (3)	0.36724 (18)	0.0363 (5)	
H5	0.3435	0.5647	0.4130	0.044*	
C6	0.2671 (2)	0.5404 (3)	0.26407 (19)	0.0380 (5)	
H6	0.2758	0.4465	0.2386	0.046*	
C7	0.2116 (2)	0.6075 (2)	0.19524 (17)	0.0332 (5)	
H7	0.1802	0.5566	0.1243	0.040*	
C8	0.2030 (2)	0.7453 (2)	0.23036 (15)	0.0263 (4)	
C9	0.2492 (2)	0.8205 (2)	0.33623 (15)	0.0258 (4)	
C10	0.2157 (2)	1.1546 (2)	0.50524 (15)	0.0295 (4)	
C11	0.0945 (2)	1.1650 (2)	0.45270 (16)	0.0323 (4)	
H11	0.0402	1.0879	0.3954	0.039*	
C12	0.0535 (3)	1.2877 (3)	0.48423 (18)	0.0404 (5)	
H12	−0.0279	1.2952	0.4476	0.049*	
C13	0.1302 (3)	1.3993 (3)	0.56847 (19)	0.0467 (6)	
H13	0.1011	1.4829	0.5901	0.056*	
C14	0.2493 (3)	1.3890 (3)	0.62121 (18)	0.0452 (6)	
H14	0.3026	1.4663	0.6788	0.054*	
C15	0.2915 (2)	1.2678 (3)	0.59103 (16)	0.0371 (5)	
H15	0.3725	1.2609	0.6287	0.045*	
C16	0.1809 (2)	0.8010 (2)	0.02145 (15)	0.0259 (4)	
C17	0.3276 (2)	0.8225 (2)	0.00430 (17)	0.0353 (5)	
H17	0.3998	0.8545	0.0622	0.042*	
C18	0.3692 (3)	0.7974 (3)	−0.09724 (19)	0.0414 (5)	
H18	0.4695	0.8110	−0.1090	0.050*	
C19	0.2635 (3)	0.7524 (3)	−0.18145 (17)	0.0399 (5)	
H19	0.2919	0.7352	−0.2510	0.048*	
C20	0.1189 (3)	0.7325 (3)	−0.16542 (16)	0.0373 (5)	
H20	0.0476	0.7019	−0.2238	0.045*	
C21	0.0756 (2)	0.7571 (2)	−0.06359 (15)	0.0294 (4)	
H21	−0.0248	0.7440	−0.0524	0.035*	
C22	−0.0677 (2)	0.7813 (2)	0.14853 (14)	0.0242 (4)	
C23	−0.1542 (2)	0.6327 (2)	0.12208 (16)	0.0332 (5)	
H23	−0.1087	0.5599	0.1105	0.040*	
C24	−0.3069 (3)	0.5900 (3)	0.11247 (18)	0.0415 (5)	
H24	−0.3660	0.4882	0.0948	0.050*	
C25	−0.3732 (2)	0.6962 (3)	0.12869 (17)	0.0387 (5)	
H25	−0.4778	0.6668	0.1215	0.046*	
C26	−0.2891 (2)	0.8431 (3)	0.15501 (16)	0.0343 (5)	
H26	−0.3355	0.9151	0.1665	0.041*	
C27	−0.1357 (2)	0.8872 (2)	0.16492 (15)	0.0283 (4)	
H27	−0.0774	0.9893	0.1828	0.034*	

### Dichlorido[8-(diphenylphosphanyl)-2-phenylquinoline-κ2N</i.,P]palladium(II) (complex2) Atomic displacement parameters (Å^2^)

**Table:**

	*U*^11^	*U*^22^	*U*^33^	*U*^12^	*U*^13^	*U*^23^
Pd1	0.02264 (9)	0.02275 (9)	0.02375 (9)	0.01079 (6)	0.00033 (5)	0.00750 (6)
Cl1	0.0271 (2)	0.0258 (3)	0.0334 (2)	0.00800 (19)	−0.00090 (17)	0.00894 (18)
Cl2	0.0398 (3)	0.0379 (3)	0.0305 (3)	0.0188 (2)	−0.00031 (19)	0.0154 (2)
P1	0.0233 (3)	0.0241 (3)	0.0258 (2)	0.0119 (2)	0.00061 (17)	0.00659 (18)
N1	0.0238 (8)	0.0290 (9)	0.0236 (8)	0.0101 (7)	0.0007 (6)	0.0094 (6)
C1	0.0221 (10)	0.0328 (11)	0.0306 (10)	0.0069 (8)	0.0027 (7)	0.0108 (8)
C2	0.0312 (12)	0.0494 (14)	0.0277 (10)	0.0127 (10)	0.0002 (8)	0.0168 (9)
C3	0.0289 (11)	0.0514 (14)	0.0353 (11)	0.0155 (10)	0.0012 (8)	0.0247 (10)
C4	0.0226 (10)	0.0373 (12)	0.0399 (11)	0.0133 (9)	0.0051 (8)	0.0205 (9)
C5	0.0278 (11)	0.0397 (13)	0.0532 (14)	0.0174 (10)	0.0063 (9)	0.0265 (10)
C6	0.0337 (12)	0.0306 (12)	0.0593 (14)	0.0185 (10)	0.0079 (10)	0.0185 (10)
C7	0.0319 (12)	0.0304 (12)	0.0416 (12)	0.0156 (9)	0.0028 (8)	0.0098 (9)
C8	0.0243 (10)	0.0252 (10)	0.0333 (10)	0.0113 (8)	0.0026 (7)	0.0103 (8)
C9	0.0202 (10)	0.0280 (10)	0.0343 (10)	0.0110 (8)	0.0044 (7)	0.0137 (8)
C10	0.0280 (11)	0.0336 (11)	0.0256 (10)	0.0073 (9)	0.0067 (7)	0.0097 (8)
C11	0.0298 (11)	0.0328 (12)	0.0333 (11)	0.0096 (9)	0.0053 (8)	0.0074 (8)
C12	0.0358 (13)	0.0418 (14)	0.0495 (13)	0.0179 (11)	0.0133 (10)	0.0148 (10)
C13	0.0548 (16)	0.0330 (13)	0.0522 (14)	0.0156 (12)	0.0187 (11)	0.0083 (10)
C14	0.0531 (16)	0.0355 (13)	0.0358 (12)	0.0040 (11)	0.0104 (10)	0.0034 (9)
C15	0.0349 (12)	0.0411 (13)	0.0290 (11)	0.0055 (10)	0.0037 (8)	0.0073 (9)
C16	0.0278 (10)	0.0238 (10)	0.0289 (10)	0.0129 (8)	0.0035 (7)	0.0054 (7)
C17	0.0281 (11)	0.0368 (13)	0.0430 (12)	0.0150 (10)	0.0037 (8)	0.0067 (9)
C18	0.0336 (13)	0.0388 (13)	0.0566 (15)	0.0172 (10)	0.0203 (10)	0.0126 (10)
C19	0.0546 (15)	0.0323 (12)	0.0373 (12)	0.0196 (11)	0.0190 (10)	0.0091 (9)
C20	0.0477 (14)	0.0367 (13)	0.0286 (11)	0.0163 (11)	0.0048 (9)	0.0069 (9)
C21	0.0299 (11)	0.0307 (11)	0.0315 (10)	0.0141 (9)	0.0045 (8)	0.0090 (8)
C22	0.0236 (10)	0.0295 (10)	0.0225 (9)	0.0111 (8)	0.0028 (7)	0.0087 (7)
C23	0.0337 (12)	0.0301 (12)	0.0393 (11)	0.0147 (9)	0.0030 (8)	0.0091 (9)
C24	0.0353 (13)	0.0332 (13)	0.0512 (14)	0.0048 (10)	0.0013 (10)	0.0115 (10)
C25	0.0241 (11)	0.0519 (15)	0.0429 (12)	0.0123 (10)	0.0031 (8)	0.0184 (10)
C26	0.0321 (12)	0.0459 (14)	0.0342 (11)	0.0230 (10)	0.0043 (8)	0.0127 (9)
C27	0.0292 (11)	0.0300 (11)	0.0288 (10)	0.0132 (9)	0.0010 (7)	0.0085 (8)

### Dichlorido[8-(diphenylphosphanyl)-2-phenylquinoline-κ2N</i.,P]palladium(II) (complex2) Geometric parameters (Å, º)

**Table:**

Pd1—N1	2.0806 (15)	C12—H12	0.9500
Pd1—P1	2.2036 (6)	C13—C14	1.380 (4)
Pd1—Cl2	2.2769 (5)	C13—H13	0.9500
Pd1—Cl1	2.3738 (6)	C14—C15	1.376 (4)
P1—C22	1.8094 (19)	C14—H14	0.9500
P1—C16	1.8112 (19)	C15—H15	0.9500
P1—C8	1.821 (2)	C16—C17	1.387 (3)
N1—C1	1.330 (2)	C16—C21	1.394 (3)
N1—C9	1.383 (3)	C17—C18	1.387 (3)
C1—C2	1.425 (3)	C17—H17	0.9500
C1—C10	1.479 (3)	C18—C19	1.387 (3)
C2—C3	1.354 (3)	C18—H18	0.9500
C2—H2	0.9500	C19—C20	1.367 (3)
C3—C4	1.423 (3)	C19—H19	0.9500
C3—H3	0.9500	C20—C21	1.396 (3)
C4—C5	1.406 (3)	C20—H20	0.9500
C4—C9	1.422 (3)	C21—H21	0.9500
C5—C6	1.370 (3)	C22—C23	1.388 (3)
C5—H5	0.9500	C22—C27	1.395 (3)
C6—C7	1.420 (3)	C23—C24	1.387 (3)
C6—H6	0.9500	C23—H23	0.9500
C7—C8	1.374 (3)	C24—C25	1.385 (3)
C7—H7	0.9500	C24—H24	0.9500
C8—C9	1.416 (3)	C25—C26	1.368 (3)
C10—C15	1.398 (3)	C25—H25	0.9500
C10—C11	1.398 (3)	C26—C27	1.392 (3)
C11—C12	1.383 (3)	C26—H26	0.9500
C11—H11	0.9500	C27—H27	0.9500
C12—C13	1.380 (3)		
			
N1—Pd1—P1	83.24 (5)	C13—C12—C11	120.5 (2)
N1—Pd1—Cl2	173.78 (5)	C13—C12—H12	119.8
P1—Pd1—Cl2	90.56 (2)	C11—C12—H12	119.8
N1—Pd1—Cl1	94.56 (5)	C12—C13—C14	119.8 (2)
P1—Pd1—Cl1	165.930 (19)	C12—C13—H13	120.1
Cl2—Pd1—Cl1	91.57 (2)	C14—C13—H13	120.1
C22—P1—C16	106.75 (9)	C15—C14—C13	120.5 (2)
C22—P1—C8	110.11 (9)	C15—C14—H14	119.7
C16—P1—C8	106.83 (9)	C13—C14—H14	119.7
C22—P1—Pd1	117.05 (7)	C14—C15—C10	120.3 (2)
C16—P1—Pd1	117.40 (7)	C14—C15—H15	119.9
C8—P1—Pd1	97.85 (7)	C10—C15—H15	119.9
C1—N1—C9	119.93 (16)	C17—C16—C21	119.90 (18)
C1—N1—Pd1	124.98 (14)	C17—C16—P1	119.09 (15)
C9—N1—Pd1	113.93 (11)	C21—C16—P1	120.78 (15)
N1—C1—C2	119.8 (2)	C18—C17—C16	120.1 (2)
N1—C1—C10	118.68 (17)	C18—C17—H17	119.9
C2—C1—C10	121.43 (18)	C16—C17—H17	119.9
C3—C2—C1	121.03 (19)	C17—C18—C19	119.6 (2)
C3—C2—H2	119.5	C17—C18—H18	120.2
C1—C2—H2	119.5	C19—C18—H18	120.2
C2—C3—C4	119.82 (18)	C20—C19—C18	120.7 (2)
C2—C3—H3	120.1	C20—C19—H19	119.7
C4—C3—H3	120.1	C18—C19—H19	119.7
C5—C4—C3	124.78 (19)	C19—C20—C21	120.2 (2)
C5—C4—C9	118.47 (19)	C19—C20—H20	119.9
C3—C4—C9	116.74 (19)	C21—C20—H20	119.9
C6—C5—C4	121.20 (19)	C16—C21—C20	119.4 (2)
C6—C5—H5	119.4	C16—C21—H21	120.3
C4—C5—H5	119.4	C20—C21—H21	120.3
C5—C6—C7	120.0 (2)	C23—C22—C27	119.44 (19)
C5—C6—H6	120.0	C23—C22—P1	122.85 (16)
C7—C6—H6	120.0	C27—C22—P1	117.58 (15)
C8—C7—C6	120.6 (2)	C24—C23—C22	120.2 (2)
C8—C7—H7	119.7	C24—C23—H23	119.9
C6—C7—H7	119.7	C22—C23—H23	119.9
C7—C8—C9	119.58 (17)	C25—C24—C23	119.9 (2)
C7—C8—P1	126.59 (15)	C25—C24—H24	120.1
C9—C8—P1	113.81 (14)	C23—C24—H24	120.1
N1—C9—C8	118.45 (16)	C26—C25—C24	120.5 (2)
N1—C9—C4	121.43 (17)	C26—C25—H25	119.8
C8—C9—C4	120.11 (18)	C24—C25—H25	119.8
C15—C10—C11	118.9 (2)	C25—C26—C27	120.2 (2)
C15—C10—C1	121.50 (19)	C25—C26—H26	119.9
C11—C10—C1	119.55 (18)	C27—C26—H26	119.9
C12—C11—C10	120.0 (2)	C26—C27—C22	119.9 (2)
C12—C11—H11	120.0	C26—C27—H27	120.1
C10—C11—H11	120.0	C22—C27—H27	120.1
			
C9—N1—C1—C2	11.7 (3)	C15—C10—C11—C12	1.6 (3)
Pd1—N1—C1—C2	−155.23 (15)	C1—C10—C11—C12	−179.87 (19)
C9—N1—C1—C10	−165.13 (17)	C10—C11—C12—C13	−1.0 (3)
Pd1—N1—C1—C10	28.0 (2)	C11—C12—C13—C14	0.5 (4)
N1—C1—C2—C3	−3.4 (3)	C12—C13—C14—C15	−0.6 (4)
C10—C1—C2—C3	173.3 (2)	C13—C14—C15—C10	1.2 (3)
C1—C2—C3—C4	−5.3 (3)	C11—C10—C15—C14	−1.7 (3)
C2—C3—C4—C5	−174.4 (2)	C1—C10—C15—C14	179.83 (19)
C2—C3—C4—C9	5.5 (3)	C22—P1—C16—C17	−172.40 (17)
C3—C4—C5—C6	178.0 (2)	C8—P1—C16—C17	−54.62 (19)
C9—C4—C5—C6	−1.8 (3)	Pd1—P1—C16—C17	53.89 (19)
C4—C5—C6—C7	−1.0 (3)	C22—P1—C16—C21	13.03 (19)
C5—C6—C7—C8	2.1 (3)	C8—P1—C16—C21	130.82 (17)
C6—C7—C8—C9	−0.3 (3)	Pd1—P1—C16—C21	−120.67 (16)
C6—C7—C8—P1	−178.71 (17)	C21—C16—C17—C18	−1.4 (3)
C22—P1—C8—C7	80.9 (2)	P1—C16—C17—C18	−175.99 (17)
C16—P1—C8—C7	−34.6 (2)	C16—C17—C18—C19	0.8 (3)
Pd1—P1—C8—C7	−156.42 (18)	C17—C18—C19—C20	0.0 (4)
C22—P1—C8—C9	−97.56 (16)	C18—C19—C20—C21	−0.1 (4)
C16—P1—C8—C9	146.89 (14)	C17—C16—C21—C20	1.2 (3)
Pd1—P1—C8—C9	25.08 (15)	P1—C16—C21—C20	175.74 (16)
C1—N1—C9—C8	167.48 (18)	C19—C20—C21—C16	−0.5 (3)
Pd1—N1—C9—C8	−24.2 (2)	C16—P1—C22—C23	66.97 (18)
C1—N1—C9—C4	−11.5 (3)	C8—P1—C22—C23	−48.63 (19)
Pd1—N1—C9—C4	156.78 (15)	Pd1—P1—C22—C23	−159.13 (14)
C7—C8—C9—N1	178.38 (19)	C16—P1—C22—C27	−108.83 (15)
P1—C8—C9—N1	−3.0 (2)	C8—P1—C22—C27	135.57 (15)
C7—C8—C9—C4	−2.6 (3)	Pd1—P1—C22—C27	25.07 (16)
P1—C8—C9—C4	176.01 (15)	C27—C22—C23—C24	−0.2 (3)
C5—C4—C9—N1	−177.37 (18)	P1—C22—C23—C24	−175.97 (16)
C3—C4—C9—N1	2.8 (3)	C22—C23—C24—C25	0.4 (3)
C5—C4—C9—C8	3.7 (3)	C23—C24—C25—C26	−0.5 (3)
C3—C4—C9—C8	−176.20 (18)	C24—C25—C26—C27	0.5 (3)
N1—C1—C10—C15	−146.2 (2)	C25—C26—C27—C22	−0.3 (3)
C2—C1—C10—C15	37.0 (3)	C23—C22—C27—C26	0.2 (3)
N1—C1—C10—C11	35.3 (3)	P1—C22—C27—C26	176.17 (15)
C2—C1—C10—C11	−141.5 (2)		
